# Deep convolutional neural network-based detection of meniscus tears: comparison with radiologists and surgery as standard of reference

**DOI:** 10.1007/s00256-020-03410-2

**Published:** 2020-03-13

**Authors:** Benjamin Fritz, Giuseppe Marbach, Francesco Civardi, Sandro F. Fucentese, Christian W.A. Pfirrmann

**Affiliations:** 1 Department of Radiology, Balgrist University Hospital, Forchstrasse 340, CH-8008 Zurich, Switzerland; 2 Faculty of Medicine, University of Zurich, Zurich, Switzerland; 3 Balzano Informatik AG, Zurich, Switzerland; 4 Department of Orthopedic Surgery, Balgrist University Hospital, Zurich, Switzerland

**Keywords:** Artificial intelligence, Neural networks (computer), Tibial meniscus injuries, Data accuracy, Magnetic resonance imaging

## Abstract

**Objective:**

To clinically validate a fully automated deep convolutional neural network (DCNN) for detection of surgically proven meniscus tears.

**Materials and methods:**

One hundred consecutive patients were retrospectively included, who underwent knee MRI and knee arthroscopy in our institution. All MRI were evaluated for medial and lateral meniscus tears by two musculoskeletal radiologists independently and by DCNN. Included patients were not part of the training set of the DCNN. Surgical reports served as the standard of reference. Statistics included sensitivity, specificity, accuracy, ROC curve analysis, and kappa statistics.

**Results:**

Fifty-seven percent (57/100) of patients had a tear of the medial and 24% (24/100) of the lateral meniscus, including 12% (12/100) with a tear of both menisci. For medial meniscus tear detection, sensitivity, specificity, and accuracy were for reader 1: 93%, 91%, and 92%, for reader 2: 96%, 86%, and 92%, and for the DCNN: 84%, 88%, and 86%. For lateral meniscus tear detection, sensitivity, specificity, and accuracy were for reader 1: 71%, 95%, and 89%, for reader 2: 67%, 99%, and 91%, and for the DCNN: 58%, 92%, and 84%. Sensitivity for medial meniscus tears was significantly different between reader 2 and the DCNN (*p* = 0.039), and no significant differences existed for all other comparisons (all *p* ≥ 0.092). The AUC-ROC of the DCNN was 0.882, 0.781, and 0.961 for detection of medial, lateral, and overall meniscus tear. Inter-reader agreement was very good for the medial (kappa = 0.876) and good for the lateral meniscus (kappa = 0.741).

**Conclusion:**

DCNN-based meniscus tear detection can be performed in a fully automated manner with a similar specificity but a lower sensitivity in comparison with musculoskeletal radiologists.

## Introduction

Meniscus tears are common findings in patients with knee pain, which in most cases are caused by trauma or degeneration [1–3]. Studies showed an association of meniscus tears with persistent knee pain, reduced function, and early osteoarthritis [4–6]. Treatment can be divided into conservative and surgical management options, depending on a variety of factors, including the shape, size, and location of the meniscus tear, as well as the age and physical activity of the patient [7–10]. Adequate treatment may reduce sequela of meniscus tears, improve quality of life, and reduce health care costs [11–14]. Therefore, accurate diagnosis of meniscus tears is important.

Owing to the high soft tissue contrast of MRI, fluid-sensitive sequences are accurate for detecting meniscal tears. In comparison with arthroscopy, MRI has a sensitivity and specificity of 93% and 88% for medial and 79% and 96% for lateral meniscus tear detection, respectively, replacing diagnostic arthroscopy in large part nowadays [15–17].

Increasing computing power and improved big data management have led to substantial advances of artificial intelligence (AI) [18, 19]. Machine learning and deep learning are subcategories of the broader field of AI, which describe concepts of self-learning computer algorithms with the capability of solving specific tasks without being programmed with explicit rules [20, 21]. In particular, great progress has been made in the field of image classification over the past decade. This progress was driven by improvements of the deep learning algorithms and graphic processing units. Algorithms based on convolutional neural networks (CNN), which today are the state-of-the-art methodology in many visual recognition tasks [22, 23], may recognize and localize objects in images with similar or even better accuracy than humans [24]. CNN compose of multiple connected layers, which each alter data and learn to detect specific image features, eventually leading to a classification output. Despite this progress, training of a CNN model is still a challenge, because the tasks are often computationally intense and require large training data sets. With multiple new MRI techniques that permit full knee MRI exams in 5–10 min, fast interpretation with the aid of AI is expected to become more and more important in order to match the efficiency of study acquisition and interpretation [25–27].

This study evaluates a deep convolutional neural network (DCNN) for detection of medial and lateral meniscus tears, which was trained on more than 18,500 MR examinations from various institutions. However, no clinical evaluation and correlation to surgical findings have been performed yet, and the DCNN’s true capabilities for meniscus tear detection in a clinical setting are unclear so far. Therefore, the purpose of this study was to clinically validate a fully automated DCNN for detection of surgically proven meniscus tears.

## Material and methods

This retrospective study was approved by our local ethics committee. Written informed consent for retrospective data analysis was obtained from all included subjects.

### Study design and participants

**Fig. 1:**
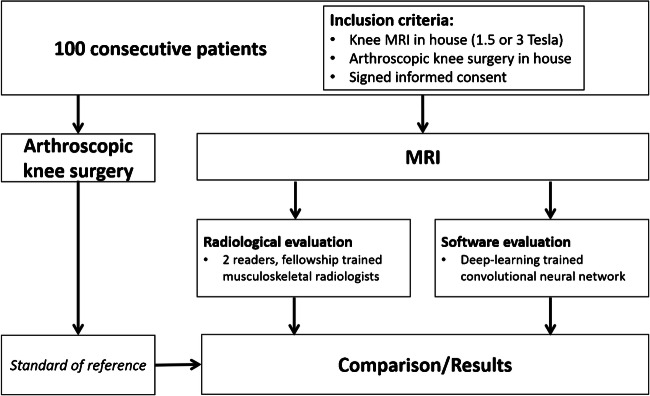
Flowchart of the study design

**Fig. 2:**
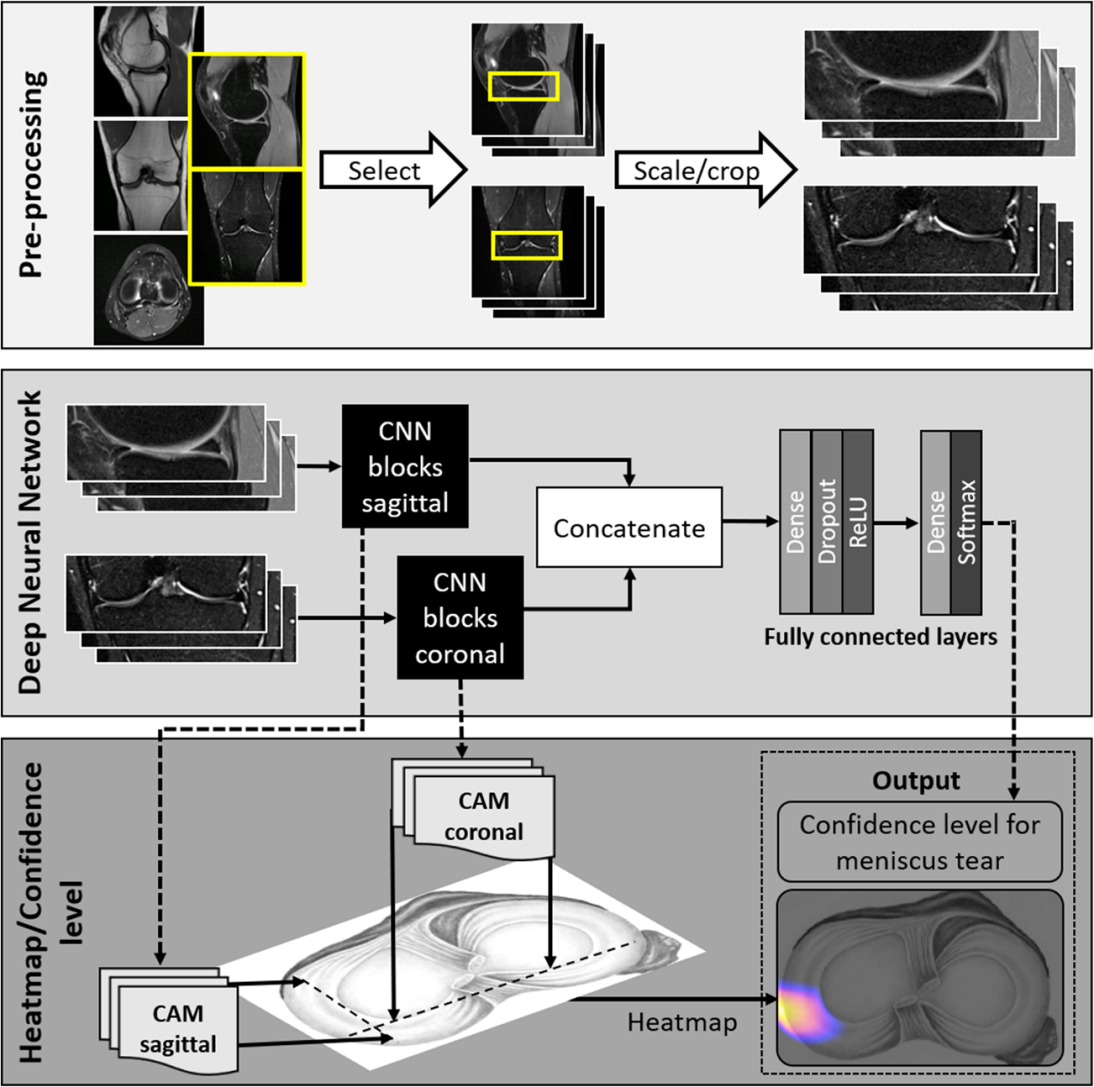
Schematic illustration of the deep learning-based software. The top box represents the initial preprocessing step. Out of a full set of sequences of a knee MR examination, the algorithm selects a coronal and a sagittal fluid-sensitive fat-suppressed sequence with subsequent rescaling and cropping around the menisci. Both sequences are the input for the deep convolutional neural network (CNN), represented by the middle box. The sagittal and coronal images are processed by two distinct convolution blocks and the results are concatenated before being processed by the dense layers. Finally, a confidence level for a tear of the medial and lateral meniscus is computed by a softmax layer within the second dense layer. The bottom box represents the localization of the meniscus tear on an axial image of both menisci, using a color-coded heatmap. Therefore, the class activation map (CAM) of the last convolutional layer of the CNN is calculated. Please note that the heatmap is still under development and was therefore not evaluated in our study. ReLU = rectified linear unit

Figure 1 presents a flowchart of the study design. Knee MRI exams of clinical patients were retrospectively evaluated by two radiologists and by a deep convolutional neural network (DCNN)-based software for detection of medial and lateral meniscus tears (Fig. 2). All included patients had undergone arthroscopic knee surgery with meniscus evaluation after the MRI. The report of the knee surgery served as the standard of reference of this study. Radiological assessments and results of the DCNN were compared, and differences of diagnostic performances were calculated.

We included 100 consecutive patients, which were referred to our institution for MRI of the knee joint by a board-certified physician for the evaluation of knee pain. The included patients were not part of the set used for training or internal validation of the DCNN and were included if the following criteria were met: (1) MRI of the knee joint performed at our institution on a clinical 1.5 Tesla or 3 Tesla clinical whole-body MRI system using our standard protocols for evaluation of knee pain (Table 1); (2) arthroscopic knee surgery performed at our institution by a specialized knee surgeon, at a time interval of less than 3 months after the knee MRI; and (3) signed informed consent for retrospective data analysis. Patients were excluded in case of previous knee surgery or impaired image quality due to motion. Knee MRI were performed between April 2016 and April 2018. The study population consisted of 46 women and 54 men with a mean age of 39.9 years (standard deviation (SD) 14.3 years; range 14–74 years). Age was not significantly different between women (mean 40.1 ± 14.2 years) and men (mean 39.7 ± 14.6 years) with *p* = 0.893. Sixty-four patients were examined on a 1.5 Tesla (T) and 36 patients were examined on a 3 T MR scanner.

**Table 1:** Standard MR imaging protocol for knee trauma at 1.5 Tesla and 3 Tesla

	Sequence	Plane	TR/TE (ms)	FOV (mm)	Slice thickness (mm)	Matrix
1.5 Tesla	T1	Cor	562/14	170 × 170	3	336 × 448
	STIR	Cor	4000/39	170 × 170	3	288 × 384
	IW fs	Tra	3600/31	160 × 160	2.5	314 × 448
	IW Dixon	Sag	3080/27	163 × 180	3	325 × 448
3 Tesla	T1	Cor	700/10	160 × 160	3	358 × 448
	STIR	Cor	4460/34	160 × 160	3	307 × 384
	IW fs	Tra	4480/40	150 × 150	2.5	307 × 384
	IW Dixon	Sag	3780/39	160 × 160	3	358 × 448

*TR* repetition time, *TE* echo time, *FOV* field of view, *Cor* coronal, *Sag* sagittal, *Tra* transverse, *STIR* short-tau inversion recovery, *IW* intermediate-weighted sequence, *Fs* fat suppression, *Dixon* Dixon technique with in-phase and fluid-sensitive sequences

### Surgical report

All surgeries were performed by board-certified specialized knee surgeons. Using standard anterolateral and anteromedial arthroscopic portals, a structured arthroscopic evaluation of all knee compartments and all intraarticular structures (i.e., menisci, ligaments, cartilage, synovitis) is performed on any procedure. Due to institutional guidelines, detailed reports of all findings were performed for each knee compartment in a standardized fashion.

### MR imaging

All patients were examined on a clinical 1.5 T or 3 T MRI system (Magnetom Avanto fit or Magnetom Skyra fit, Siemens Healthcare, Erlangen, Germany) with a dedicated 15 channel transmit/receive knee coil. All examinations consisted of a coronal T1-weighted, coronal short-tau inversion recovery (STIR), axial fat-suppressed intermediate weighted (IW), and sagittal fat-suppressed and nonfat-suppressed IW sequences, acquired in Dixon technique (in-phase and water-only images). Parameters for the 1.5 T and 3 T protocol are given in Table 1.

### Meniscus evaluation

Knee MRI of all patients were separately evaluated by two full-time and fellowship-trained musculoskeletal radiologists (reader 1: BF, 7 years of experience in musculoskeletal radiology; reader 2: CP, 21 years of experience in musculoskeletal radiology). Evaluations were performed on anonymized data sets after removal of any personal or clinical information on a state-of-the-art picture archiving and communication system (PACS) workstations (MERLIN Diagnostic Workcenter, version 5.2, Phönix-PACS GmbH, Freiburg, Germany) in radiological reading room conditions. Both readers were blinded to the patients’ clinical histories, intraoperative findings, or the indications for knee surgery.

**Fig. 3:**
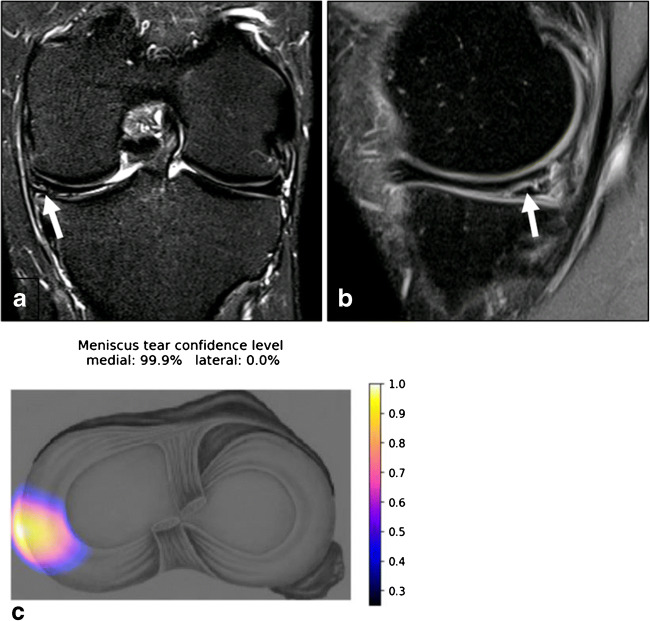
MRI of the left knee joint of a 30-year-old male patient. **a** A coronal short-tau inversion recovery image of the body of both menisci. **b** A sagittal fat-suppressed intermediate-weighted image at the junction of the posterior horn to the body of the medial meniscus. **c** The output of the deep convolutional neural network, which calculates a probability of a tear of the medial and lateral meniscus as well as provides a heatmap depicting the location of the suspected tear. A horizontal meniscus tear is present at the body with extension to the posterior horn to the medial meniscus (arrows). Knee arthroscopy confirmed the tear of the medial meniscus. Both readers and the deep convolutional neural network diagnosed the medial meniscus tear correctly; the probability of a tear was estimated with 99.9% by the deep convolutional neural network

**Fig. 4:**
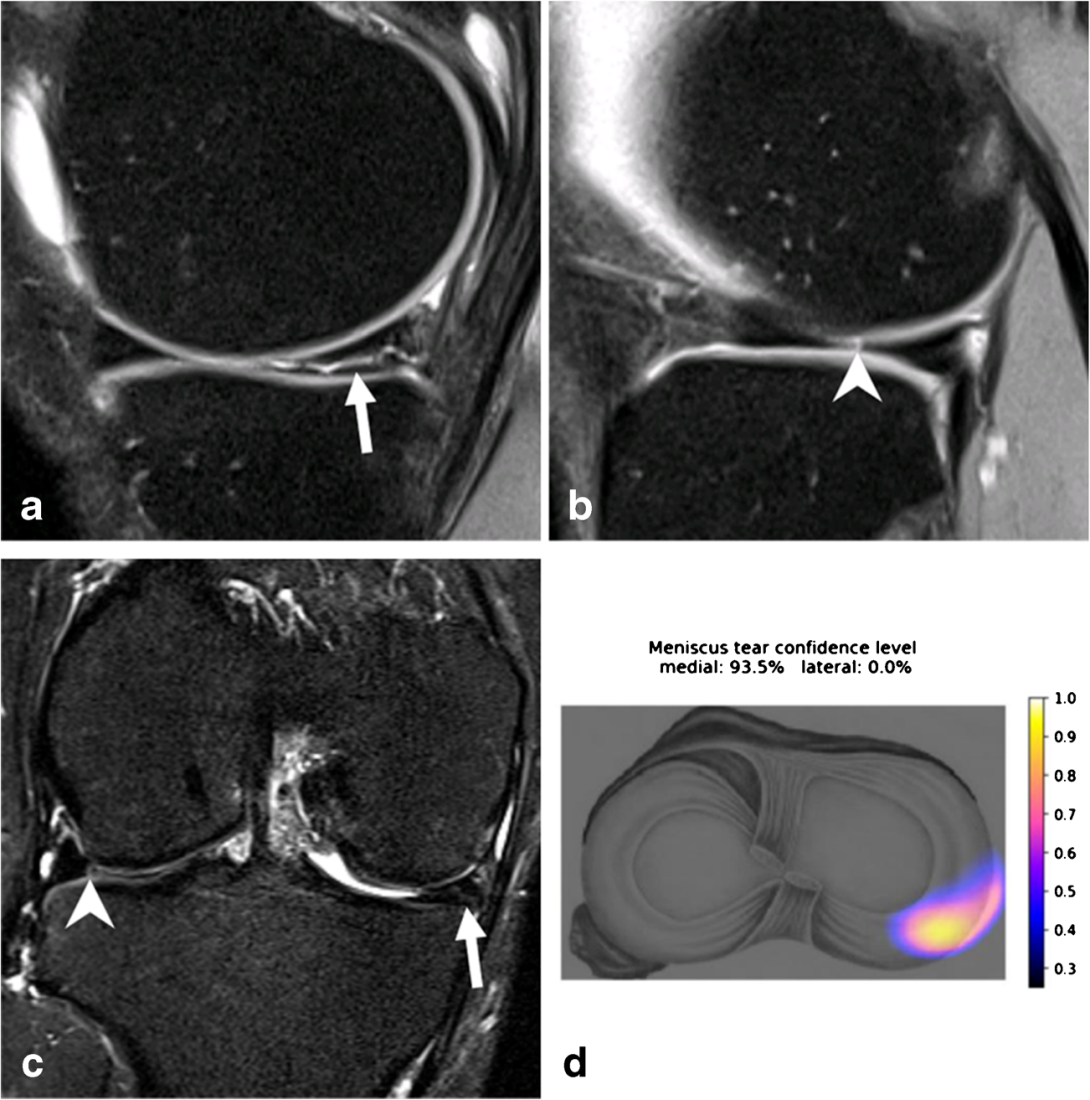
MRI of the right knee joint of a 45-year-old male patient. Sagittal fat-suppressed intermediate-weighted images at the junction of the body to the posterior horn of the medial meniscus (**a**) and the lateral meniscus (**b**). **c** A coronal short-tau inversion recovery image of the posterior horns of both menisci. **d** The output of the deep convolutional neural network, which calculates a probability of a tear of the medial and lateral meniscus as well as provides a heatmap depicting the location of the suspected tear. A horizontal meniscus tear is present at the junction of the posterior horn to the body of the medial meniscus (arrows), while the lateral meniscus shows a small tear of the central body (arrowhead). Knee arthroscopy confirmed the tear of both menisci, which was correctly diagnosed by both readers. The deep convolutional neural network correctly classified the medial meniscus tear with a probability of 93.5% but missed the small tear of the lateral meniscus

For each patient, the medial and lateral meniscus was separately evaluated for the presence or absence of a meniscus tear (Figs. 3 and 4).

### Deep convolutional neural network

The deep convolutional neural network (DCNN) consists of two main components: preprocessing of the MR images—during which images are normalized to a predefined standard—and a predictive component—which computes the confidence level for the existence of meniscus tear in the knee that is depicted in the submitted MR study. The predictive part consists of a DCNN that was trained on a large proprietary database of knee MR images (Fig. 2).

#### Preprocessing

During the preprocessing stage, the DCNN automatically selects the coronal and sagittal fluid-sensitive fat-suppressed sequence, like short-tau inversion recovery or intermediate-weighted sequences. Images are then scaled to a standard pixel size, slice distance, and slice numbers using spline 3rd order interpolation. Finally, the images are cropped around the meniscus in order to reduce memory and time needed to process the convolutional neural network (CNN).

#### Convolutional neural network

The CNN receives as input the coronal and sagittal sequences and computes both planes in parallel. Each CNN block (coronal and sagittal) consists of two series of 3D convolution layers, batch-normalization layers, rectified linear unit (ReLU) activation layers and at the end of these two series of layers, one pooling layer is added. Then, four inception modules [28] preceded each by a 3D convolution layer, batch-normalization layer, and a ReLu activation layer complete the block. Each inception module ended with a pooling layer. Before concatenating the results of the two CNN blocks, the feature maps are averaged slice by slice. The network ends with two dense (or fully connected) layers: the first with a dropout and a ReLU activation layer, and the second with a softmax activation layer, which extracts the confidence level for the meniscus tear.

#### Localization (heat map)

To visually localize the tear, the software computes the class activation map (CAM) of the last convolution layer in the CNN and maps it to an axial knee image. The mapped CAM values are then scaled to the confidence level predicted by the DCNN and are represented as a heat map on an axial knee image (Figs. 3 and 4).

#### Training of the CNN

To train the CNN for detection of meniscus tears, a database of 20,520 MRI studies that met the preprocessing criteria was used: 18,520 studies were used for training, 1000 for validation, and 1000 for testing the CNN. All three data sets consisted of a pair of coronal and sagittal sequences with balanced labels (same number of knees with a torn and intact meniscus in each data set). The first data set was used to train the model (to compute the weights of the DCNN model), the second data set was used to tune the hyperparameters of the DCNN model, and finally the third data set was used as assessment of the model accuracy. The used data sets consisted of knee MRI of numerous institutions and were therefore heterogenous in terms of MR-sequence parameters and field strength. Manufacturers of the MR scanners were GE Healthcare, Waukesha, WI, USA; Philips Healthcare, Best, The Netherlands; and Siemens Healthcare, Erlangen, Germany. The data set consisted of knee MRI acquired between 2013 and 2018. The training task was performed with a binary cross entropy loss function. Adam with a learning rate of 0.001 was chosen as an optimizer to train the CNN. To develop the CNN, the Keras framework on the TensorFlow backend (keras.io and www.tensorflow.org) was used. Training was performed on an NVIDIA P-40 graphic processing unit with a batch size of 10 (studies).

#### Label extraction

The ground truths (binary labels) used to train the CNN were extracted from human-produced, anonymized clinical reports belonging to the MRI studies, using a rule-based natural language processing (NLP) algorithm. The F_1_ score of the binary label extraction was 0.97, based on 400 manually extracted labels.

### Statistics

Statistical analysis was performed using MedCalc version 17.6 (MedCalc Software bvba). General descriptive statistics were applied, and continuous data were reported as means and standard deviations and categorical data as proportions. Patient age was compared with the two-tailed independent Student’s *t* test. Sensitivity, specificity, and accuracy were calculated for radiological and DCNN assessments in comparison with the intraoperative findings and were compared using the McNemar test. Therefore, the DCNN’s probabilities for the appearance of a meniscus tear were dichotomized into present/absent using a threshold of 0.5. Using the probabilities for meniscus tears of the DCNN, receiver operating characteristic (ROC) curve analyses with calculation of the area under the ROC curves (AUC) with 95% confidence intervals (CI) were performed. Graphical visualization of results of the medial and the lateral meniscus was performed using a Zombie plot [29]. Inter-reader agreement was assessed with Cohen’s kappa. Kappa values were considered to represent good agreement if > 0.6–0.8 and excellent agreement if > 0.8–1 [30]. Subgroup analyses comparing 1.5 T and 3 T examinations were performed with Fisher’s exact test. A *p* value of < 0.05 was considered to represent statistical significance.

## Results

Fifty-seven percent (57/100) of patients had a tear of the medial meniscus and 24% (24/100) had a tear of the lateral meniscus, including 12% (12/100) of patients, who had a tear of both menisci. Thirty-one percent (31/100) of patients did not have a meniscus tear.

**Fig. 5:**
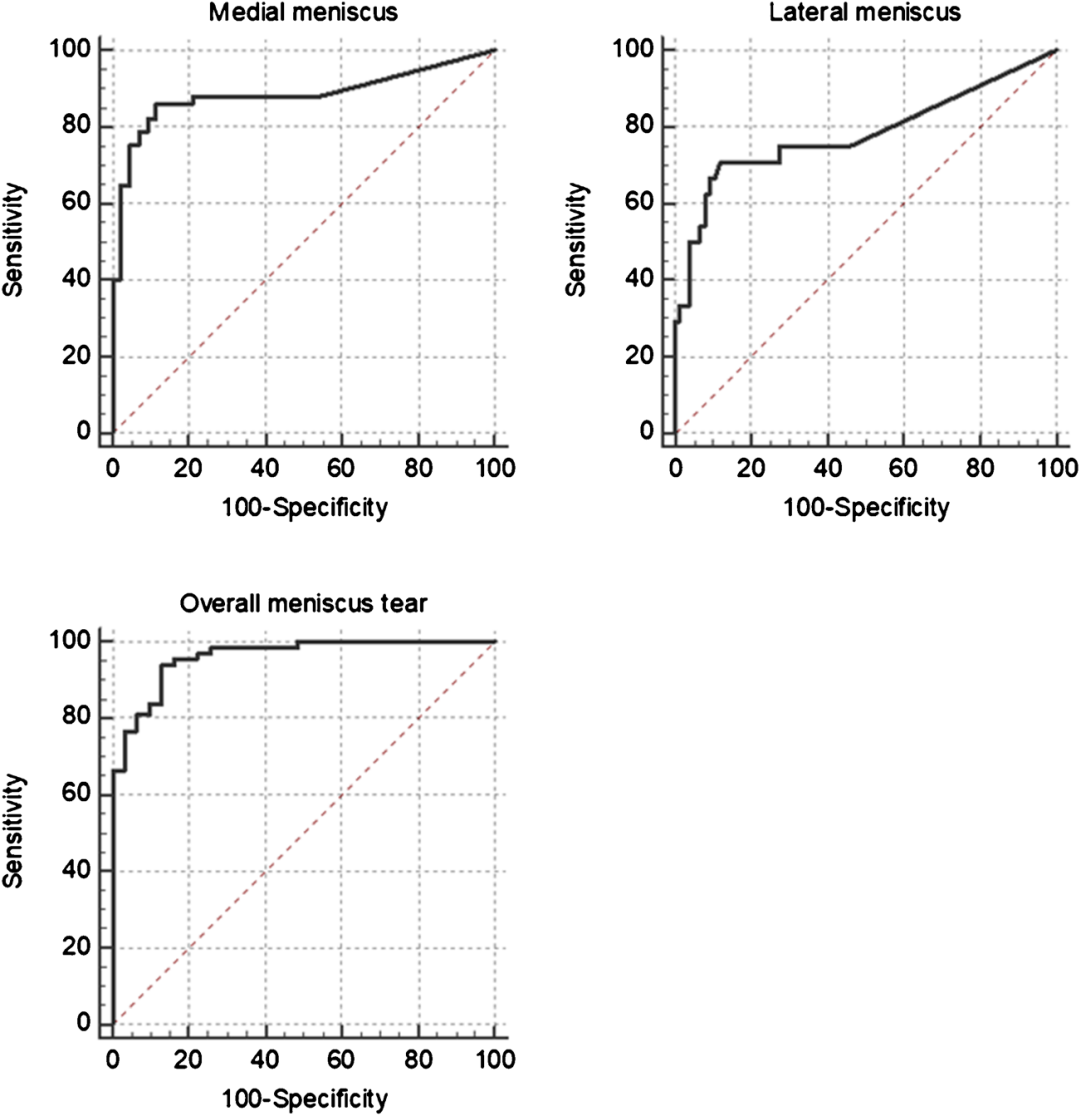
ROC curves of the deep convolutional neural network’s probabilities for a medial, lateral, and overall meniscus tears. The areas under the ROC curves (AUCs) were 0.882 (95% confidence interval (CI) 0.802; 0.938), 0.781 (95% CI 0.687, 0.858), and 0.961 (95% CI 0.902, 0.990), respectively

**Fig. 6:**
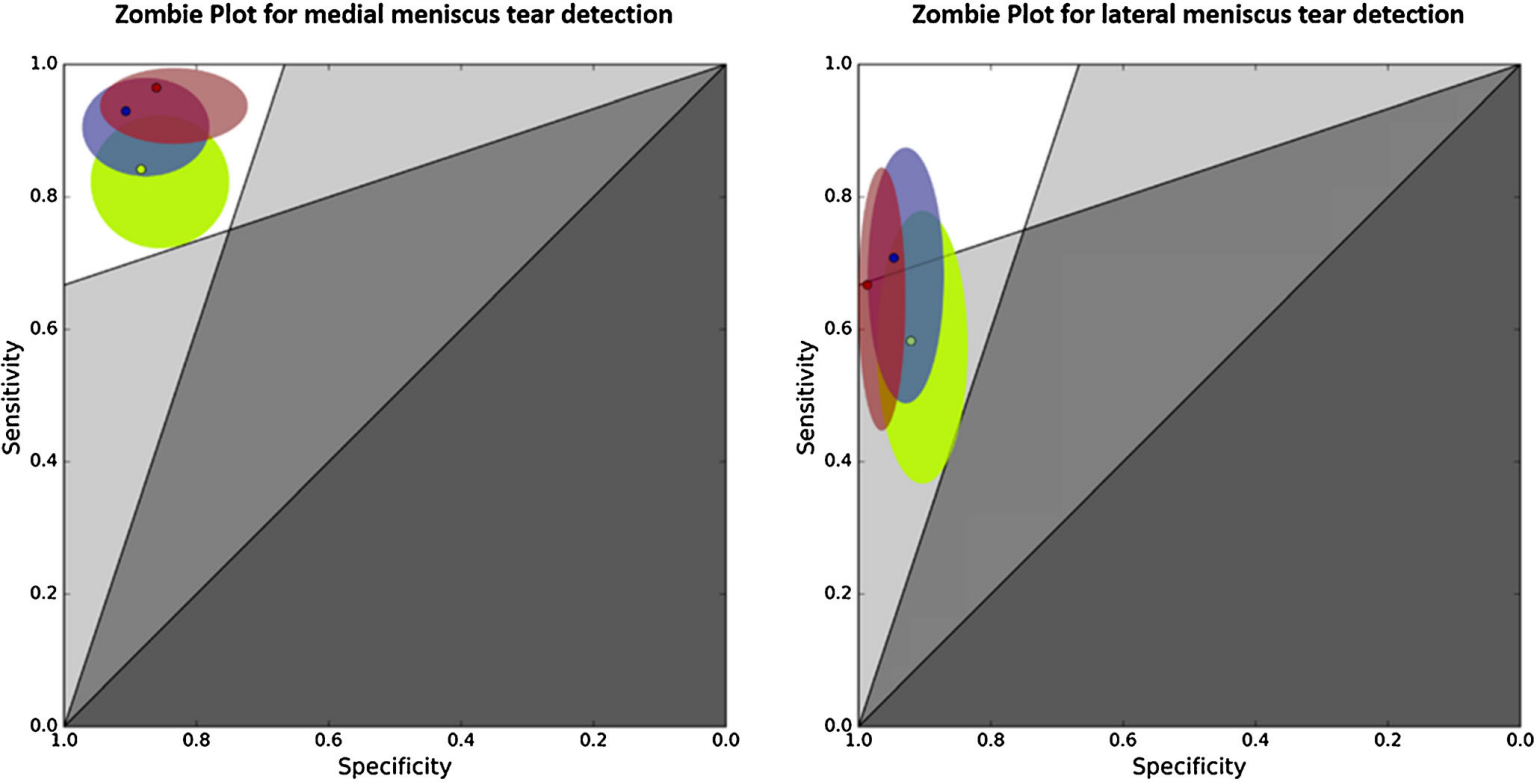
Zombie plots for graphical visualization of the estimates (solid dots) and confidence intervals of sensitivity and specificity (ellipses) of the DCNN (green), reader 1 (blue) and reader 2 (red) for medial and lateral meniscus tear detection

Table 2 and Fig. 5 show the sensitivities, specificities, accuracies, and AUCs of both readers and the DCNN for detection of medial meniscus tear, lateral meniscus tear, and global meniscus tear (including medial, lateral, or a tear of both menisci). Statistically significant differences existed only for the sensitivities for detection of a medial meniscus tear between reader 2 and the DCNN with *p* = 0.039. For all other comparisons, no significant differences existed for the medial meniscus (all *p* ≥ 0.146), the lateral meniscus (*p* ≥ 0.092), or both menisci combined (all *p* ≥ 0.344). Graphical visualization using a Zombie plot demonstrates that the results of the DCNN were centered in the “optimal zone” (upper left zone) for the medial meniscus and in the “acceptable zone for ruling in disease” for the lateral meniscus (Fig. 6) [29]. However, the results of reader 1 and reader 2 were located closer to the upper left corner, suggesting superior performance in comparison with the DCNN (Fig. 6).

**Table 2:** Results of both readers and of the deep convolutional neural network for evaluation of meniscus tears

	Medial meniscus tear				Lateral meniscus tear				Overall meniscus tear			
	Sensitivity (95% CI)	Specificity (95% CI)	Accuracy (95% CI)	AUC (95% CI)	Sensitivity (95% CI)	Specificity (95% CI)	Accuracy (95% CI)	AUC (95% CI)	Sensitivity (95% CI)	Specificity (95% CI)	Accuracy (95% CI)	AUC (95% CI)
Reader 1	93.0% (83.0%; 98.1%)	90.7% (77.9%; 97.4%)	92% (84.8%; 96.5%)	0.918 (0.846; 0.964)	70.8% (48.9%; 87.4%)	94.7% (87.1%; 98.6%)	89% (81.2%; 94.4%)	0.828 (0.739; 0.896)	94.1% (85.6%; 98.4%)	87.1% (70.2%; 96.4%)	92% (84.8%; 96.5%)	0.906 (0.832; 0.956)
Reader 2	96.5%* (87.9%; 99.6%)	86.1% (72.1%; 94.7%)	92% (84.8%; 96.5%)	0.913 (0.839; 0.960)	66.7% (44.7%; 84.4%)	98.7% (92.9%; 100.0%)	91% (83.6%; 95.8%)	0.827 (0.738; 0.895)	94.1% (85.6%; 98.4%)	93.6% (78.6%; 99.2%)	94% (87.4%; 97.8%)	0.939 (0.872; 0.977)
DCNN	84.2%* (72.1%; 92.5%)	88.4% (74.9%; 96.1%)	86% (77.6%; 92.1%)	0.882 (0.802; 0.938)	58.3% (36.6%; 77.9%)	92.1% (83.6%; 97.1%)	84% (75.3%; 90.6%	0.781 (0.687; 0.858)	91.2% (81.8%; 96.7%)	87.1% (70.2%; 96.4%)	90% (82.4%; 95.1%)	0.961 (0.902; 0.990)

A statistically significant difference existed for the sensitivities for detection of a medial meniscus tear between reader 2 and the DCNN with *p* = 0.039 (asterisk). No significant differences existed for the other comparisons. Italicized values are significantly different with *p* = 0.039

*AUC* area under the receiver operating characteristics (ROC) curve, *CI* confidence interval, *DCNN* deep convolutional neural network

Detailed analysis of the medial meniscus evaluations showed that the DCNN had 5 false positive findings (FP) and 9 false negative findings (FN). In 80% (4/5) of the FP, at least 1 reader had also an FP. In 33% (3/9) of the FN, at least 1 reader had also an FN. For reader 1, 75% (3/4) of the FP and 50% (2/4) of the FN were also rated as FP or FN by the DCNN, respectively. For reader 2, 67% (4/6) of the FP and 50% (1/2) of the FN were also rated as FP or FN by the DCNN, respectively.

Detailed analysis of the lateral meniscus evaluations showed that the DCNN had 6 FP and 10 FN. In 17% (1/6) of the FP, at least 1 reader had also an FP. In 80% (8/10) of the false negative findings, at least 1 reader had also an FN and in 50% (5/10), both readers had an FN. For reader 1, 25% (1/4) of the FP and 100% (7/7) of the FN were also rated as FP or FN by the DCNN, respectively. For reader 2, 0% (0/1) of the FP and 63% (5/8) of the FN were also rated as FP or FN by the DCNN, respectively.

Comparison of 1.5 T and 3 T examinations did not show any significant differences of sensitivities, specificities, or accuracies for any radiologist or the DCNN for neither medial (all ≥ 0.463), lateral (all ≥ 0.243), nor global meniscus tear evaluation (all ≥ 0.166).

Between both readers, the inter-reader agreement was very good for detection of medial meniscus tears with a kappa value of 0.876 (95% confidence interval 0.78; 0.972) and good for detection of lateral meniscus tears with a kappa value of 0.741 (95% confidence interval 0.572; 0.910). The kappa value for the detection of a meniscus tear in general was very good with 0.816 (95% confidence interval 0.695; 0.938).

## Discussion

In our study, we demonstrated the capability of a deep convolutional neural network (DCNN) for detection of medial and lateral meniscus tears. The DCNN’s sensitivities, specificities, and accuracies ranged between 84 and 92% for detection of medial and lateral meniscus tears except for the sensitivity for lateral meniscus tear detection, which was considerably lower with 58%. The DCNN’s sensitivity of medial meniscus tear detection was significantly lower in comparison with one of the radiologists; for all other comparisons, no significant differences existed between the DCNN and both readers.

So far, several studies have successfully implemented machine learning- or deep learning-based algorithms on clinically oriented radiological tasks. In musculoskeletal radiology, various radiograph-based tasks like bone age determination or fracture diagnosis could be demonstrated with AI-based algorithms [31, 32]. Furthermore, the feasibility of AI-based meniscus tear detection on single MRI slices on fluid-sensitive images has been demonstrated recently [33, 34]. However, AI-trained software algorithms that successfully evaluate full sets of cross-sectional imaging studies in musculoskeletal radiology are sparse so far. This might be due to several reasons. The amount of data of cross-sectional imaging is usually a multiple of the data of radiographs. A knee MR examination usually consists of about 50 megabytes of data. Considering that often thousands of studies are required for adequate training, a huge amount of data needs to be handled and vast computing capacities are required. Furthermore, MR studies consist of several sequences of different weightings and often various orientations. Findings are frequently visible on only certain sequences or weightings, and findings need to be cross-referenced between imaging planes to increase confidence levels and reach the appropriate diagnosis. The DCNN of our study overcomes some of these problems by first selecting only fluid-sensitive fat-suppressed sequences in the coronal or sagittal plane and by cropping the images to a smaller field of view, which still contains the meniscus but excluding other irrelevant structures. Similar to our study, another publication demonstrated the feasibility of a deep leaning-based algorithm for evaluation of knee MRI for meniscus tears, anterior cruciate ligament tears, and other general knee abnormalities [35]. For overall meniscus tear detection, the authors reached a sensitivity of 74.1%, a specificity of 71.0%, and an accuracy of 72.5%, which were below of our results by 16–17% for all assessments. However, a notable difference between the published and our study exists regarding the standard of reference, which limits comparability. While our study used surgical correlation as the standard of reference, the study of Bien et al. established a standard of reference by consensus of three radiologists [35]. Furthermore, our DCNN calculates a probability for a medial or lateral meniscus tear separately, while the study of Bien et al. provided only an overall probability of the occurrence of a meniscus tear. Nevertheless, the study of Bien et al. as well as our study demonstrates the capability of deep learning-trained software algorithms for detection of knee abnormalities. This is also underlined by another recent study of Liu et al., which compared the capability of a fully automated deep learning-based algorithm for detection of cartilage abnormalities on sagittal fat-suppressed T2-weighted images [36]. The performance of the deep learning-based algorithm was comparable with the performance of radiologists of different experience levels, which ranged from residents to experts. Taking also into consideration that automated segmentation of the meniscus and knee joint has become feasible [37–39], all of these studies suggest that a fully automated evaluation of the entire knee including all compartments and major structures seems to be possible in the future.

In our study, both readers showed a similar sensitivity and specificity for medial meniscus tear detection in comparison with systematic reviews, which reported pooled sensitivities of 93% and 89% and pooled specificities of 88% each, respectively [17, 40]. While the DCNN’s specificity for the medial meniscus was comparable with both readers, its sensitivity was notably lower. This difference was statistically significant in comparison with reader 2. However, due to a mildly higher specificity of the DCNN, no significant differences existed for the accuracies. However, graphical Zombie plot visualization indicated that the results of reader 1 and reader 2 were located closer to the upper left corner, suggesting superior performance in comparison with the DCNN.

A similar trend existed for the lateral meniscus. While the specificities of both readers and the DCNN were at the same level, the sensitivity of the DCNN was lower by about 10% in comparison with the readers. No statistical differences existed, which possibly relates to a low statistical power of our study since only 24 patients had a tear of the lateral meniscus. The Zombie plot demonstrated that the DCNN’s results of the lateral meniscus were mostly located in the “acceptable zone for ruling in a disease” suggesting that the DCNN is capable of ruling in a meniscus tear but also possesses an inferior performance in comparison with reader 1 and reader 2 [29]. It is remarkable that the sensitivities for lateral meniscus tear detection were overall quite low for both, the radiologists and the DCNN. Yet, systematic reviews also report a lower sensitivity for detection of lateral meniscus tears with 79% and 78%, which is well below the pooled sensitivities for medial meniscus tear detection of 93% and 89% [17, 40]. Considering that both readers of our study were full-time musculoskeletal radiologists and demonstrated good inter-reader agreement, it seems likely that some of the lateral meniscus tears were just not appreciable on MR images and were probably therefore also missed by DCNN. This assumption is supported by the large overlap of the DCNN’s false negative evaluations with the radiologists, since 80% (8/10) were also misdiagnosed as false negative by at least one reader.

An important difference exists for the evaluated sequences between the DCNN and the radiologists, which may have influenced the study results. For the meniscus assessments, the readers used the full set of knee MRI sequences, while the DCNN used only the coronal STIR sequence and the sagittal fat-suppressed IW sequence. The additional coronal T1-weighted, axial fat-suppressed IW, and the sagittal IW sequence used by the readers can offer additional diagnostic value for meniscal tear detection and may have therefore positively influenced the performance of both readers. The ground truth is another factor, which may explain in parts the lower sensitivity of the DCNN in comparison with radiologists. The ground truth was established by extracting radiologists’ diagnoses from MRI reports using NLP. While this is common practice for label extraction of large data sets, erroneous radiological interpretations introduce errors, which may negatively influence the diagnostic accuracy of the DCNN. On the other hand, an F1 score of 0.97 indicates a high accuracy of our NLP algorithm. Therefore, we believe that the influence of NLP errors was small.

The evaluated DCNN is the first step of fully automated meniscus assessment, which is a clinically important task, since meniscus tears are frequently treated with arthroscopic knee surgery [7]. While the DCNN’s performance for meniscus tear detection was close to humans, correct determination of the exact tear location is still under development. The software version, which was subject to this study, provides besides probabilities for medial and lateral meniscus tears additional heatmaps, pinpointing the exact tear location on a two-dimensional axial image by assigning each pixel a color-coded probability. However, this feature is still under development and was therefore not specifically evaluated in our study. It would also be desirable, if future developments were able to exactly characterize tear morphology and localization in terms of a periphery or center of the meniscus, which is important for determination of the adequate treatment in terms of conservative versus surgical treatment or to determine between partial meniscectomy and suture.

Our study has limitations. First, the deep learning-based DCNN was only tested on knee MRI performed at our institution. Therefore, the results of this study apply to knee examinations using our standard knee protocol and MR scanner. However, the DCNN was not fitted to our knee MR examinations but was trained on more than 18,500 knee MRI from a variety of institutions and therefore including various MR protocols and MR scanners from all major vendors and different field strengths. Therefore, we assume that the performance of the DCNN will be similar for evaluation of knee MRI of other institutions. Second, the indication for arthroscopic knee surgery was influenced by the MRI and the visibility of a meniscus tear to some degree. Still, knee arthroscopy was also performed for several other intraarticular reasons, like ligament, cartilage, or synovial abnormalities. Therefore, the study population has a relevant number of intact medial and lateral menisci; however, verification bias may be present [41].

In conclusion, DCNN-based meniscus tear detection can be performed in a fully automated manner with a similar specificity but lower sensitivity in comparison with musculoskeletal radiologists.
