# Correction to: Deep convolutional neural network-based detection of meniscus tears: comparison with radiologists and surgery as standard of reference

**DOI:** 10.1007/s00256-020-03458-0

**Published:** 2020-05-13

**Authors:** Benjamin Fritz, Giuseppe Marbach, Francesco Civardi, Sandro F. Fucentese, Christian W.A. Pfirrmann

**Affiliations:** 1 Department of Radiology, Balgrist University Hospital, Forchstrasse, 340, CH-8008 Zurich, Switzerland; 2 Faculty of Medicine, University of Zurich, Zurich, Switzerland; 3 Balzano Informatik AG, Zurich, Switzerland; 4 Department of Orthopedic Surgery, Balgrist University Hospital, Zurich, Switzerland

**Correction to: Skeletal Radiology**

**https://doi.org/10.1007/s00256-020-03410-2**

The article “Deep convolutional neural network-based detection of meniscus tears: comparison with radiologists and surgery as standard of reference”, written by Benjamin Fritz & Giuseppe Marbach & Francesco Civardi & Sandro F. Fucentese & Christian W.A. Pfirrmann, was originally published electronically on the publisher’s internet portal (currently SpringerLink) on 13 March 2020 without open access.

With the author(s)’ decision to opt for Open Choice the copyright of the article changed on April 30, 2020 to © The Author(s) 2019 and the article is forthwith distributed under the terms of the Creative Commons Attribution 4.0 International License (http://creativecommons.org/licenses/by/4.0/), which permits use, duplication, adaptation, distribution and reproduction in any medium or format, as long as you give appropriate credit to the original author(s) and the source, provide a link to the Creative Commons license and indicate if changes were made.

